# Human milk oligosaccharide 2'-fucosyllactose promotes melanin degradation via the autophagic AMPK–ULK1 signaling axis

**DOI:** 10.1038/s41598-022-17896-4

**Published:** 2022-08-17

**Authors:** Hyojin Heo, Byungsun Cha, Dongmin Jang, Chaewon Park, Gunwoo Park, Byeong-Mun Kwak, Bum-Ho Bin, Ji-Hwan Park, Mi-Gi Lee

**Affiliations:** 1 Department of Applied Biotechnology, Ajou University, Suwon, 16499 Republic of Korea; 2 Department of Biological Sciences, Ajou University, 206 Worldcup-ro, Yeongtong-gu, Suwon, 16499 Republic of Korea; 3 Department of Biomedical Sciences, Ajou University Graduate School of Medicine, Suwon, 16499 Republic of Korea; 4 Korea Bioinformation Center, Korea Research Institute of Bioscience and Biotechnology, Daejeon, 34141 Republic of Korea; 5 School of Cosmetic Science and Beauty Biotechnology, Semyung University, Chungbuk, 27136 Republic of Korea; 6 Gyeonggido Business and Science Accelerator, 107 Gwanggyo-ro, Yeongtong-gu, Suwon, 16229 Republic of Korea

**Keywords:** Biomaterials, Autophagy, Disease prevention

## Abstract

There is still an unmet need for development of safer antimelanogenic or melanin-degrading agents for skin hyperpigmentation, induced by intrinsic or extrinsic factors including aging or ultraviolet irradiation. Owing to the relatively low cytotoxicity compared with other chemical materials, several studies have explored the role of 2'-fucosyllactose (2'-FL), the most dominant component of human milk oligosaccharides. Here, we showed that 2'-FL reduced melanin levels in both melanocytic cells and a human skin equivalent three-dimensional in vitro model. Regarding the cellular and molecular mechanism, 2'-FL induced LC3I conversion into LC3II, an autophagy activation marker, followed by the formation of LC3II^+^/PMEL^+^ autophagosomes. Comparative transcriptome analysis provided a comprehensive understanding for the up- and downstream cellular processes and signaling pathways of the AMPK–ULK1 signaling axis triggered by 2'-FL treatment. Moreover, 2'-FL activated the phosphorylation of AMPK at Thr172 and of ULK1 at Ser555, which were readily reversed in the presence of dorsomorphin, a specific AMPK inhibitor, with consequent reduction of the 2'-FL-mediated hypopigmentation. Taken together, these findings demonstrate that 2'-FL promotes melanin degradation by inducing autophagy through the AMPK–ULK1 axis. Hence, 2'-FL may represent a new natural melanin-degrading agent for hyperpigmentation.

## Introduction

Several factors contribute for the abnormal activation of melanogenesis^1^, such as ultraviolet irradiation or aging, with high melanin levels causing skin problems including hyperpigmentation (or solar lentigo) and melasma. Therefore, maintaining melanin levels within the proper range is important for skin health. Different elements involved in de novo melanogenesis have attracted significant attention for the development of antimelanogenic agents^2^. Unfortunately, to date, most widely used agents are often accompanied by adverse effects, such as cytotoxicity and vitiligo-like symptoms^3,4^. Thus, development of safer antimelanogenic or melanin-degrading agents, and therapeutic regimens for hyperpigmentation is warranted for cosmetic and medical applications.

Melanin levels can be regulated by modulating either melanogenic or melanin-degrading pathways. Downregulation of melanogenesis-related intracellular signals has been suggested as an alternative strategy with therapeutic potential rather than directly targeting elements involved in melanin synthesis^5^. Noteworthy, recent advances showed that autophagy can mediate melanosome degradation in keratinocytes^6^. Indeed, chemically induced autophagy was shown to inhibit hyperpigmentation induced by the α-melanocyte-stimulating hormone^7^. Moreover, keratinocytes were found to regulate melanosome degradation via autophagy to prevent melanosome-generated toxicity^8^.

The autophagic process in eukaryotic cells involves lysosomal degradation of proteins and organelles, and is induced by cellular stress mechanisms^9–12^. Increasing evidence demonstrates that constitutive autophagy is critical for cellular homeostasis^13^ and development^14^, with autophagy deregulation contributing to various disorders^15,16^. The core autophagic molecules belong to the family of autophagy-related (Atg) proteins, including serine/threonine protein kinase Atg1 that is essential in the early stages of autophagy^12,17,18^. In particular, Unc-51-like autophagy activating kinase 1 (ULK1), the mammalian homolog of the yeast Atg1, can be phosphorylated by both the target of rapamycin (mTOR) kinase and adenosine monophosphate activated protein kinase (AMPK), but with opposite effects. AMPK is known to induce autophagy by directly phosphorylating ULK1 at Ser317, Ser777, and Ser555^19,20^, which in turn forms a stable complex with mAtg13 and FIP200 (FAK family kinase-interacting protein of 200 kDa)^19,21^; whereas mTOR inhibits AMPK-mediated ULK1 activation by phosphorylating it at Ser757^20^.

Human milk oligosaccharides are naturally produced sugars that are widely used for moisturizing the skin. Owing to their relatively low cytotoxicity compared with other chemical materials, these compounds represent promising and safe agents. In this study, we focused on 2'-fucosyllactose (2'-FL), a major constituent of the human breast milk, to explore its potential as a noncytotoxic and melanin-degrading agent. 2'-FL is expected to be a functional component not only due to its beneficial activity for human health^22–24^, but also its potential for mass production supported by microbial biosynthesis^25^. Although several studies have demonstrated the antiinflammatory activity of 2'-FL^26–28^, there are limited data on the benefits of 2'-FL to the human skin. Herein, we examined the melanin-degrading effects of 2'-FL on human and mouse melanocytes, melanoma cells, and in a human skin three-dimensional (3D) model.

## Results

### 2'-Fucosyllactose reduces melanin production

**Figure 1:**
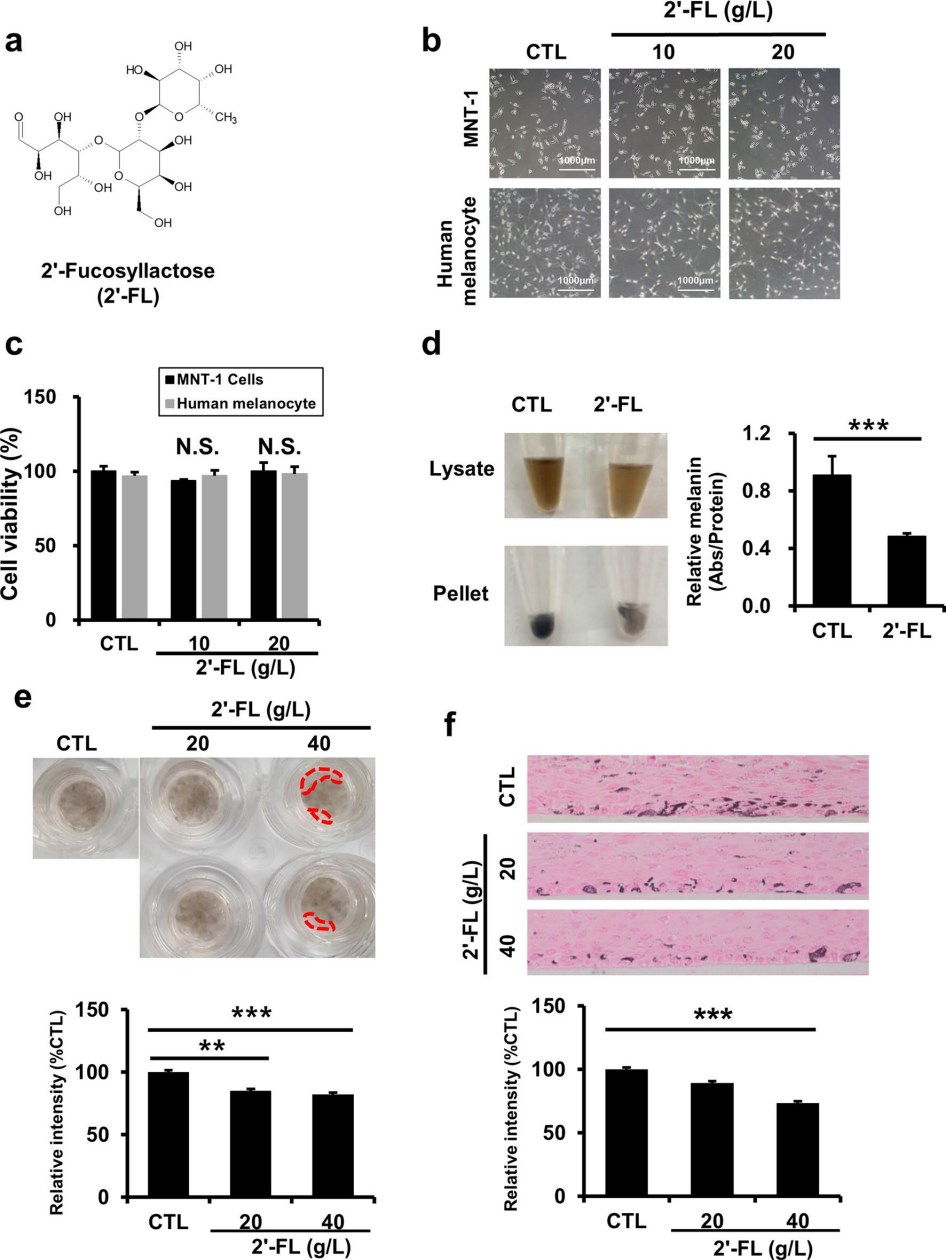
2'-Fucosyllactose reduces melanin production. (**a**) Chemical structure of 2'-FL. (**b**) Cell morphology of human melanocytes and MNT-1 cells without or with 2'-FL (10 or 20 g/L) for 24 h. (**c**) Cell viability of human melanocytes and MNT-1 cells treated with 2'-FL (10 or 20 g/L) for 24 h measured by MTT assays. Cell viability is presented in relation to nontreated cells. (**d**) Melanin levels in mouse B16 cells with or without 2'-FL (20 g/L) for 7 days. Optical densities (450 nm) are presented in relation to nontreated cells. Data in (**c**) and (**d**) are represented as mean ± SD of three replicates. *P < 0.05, ***P < 0.001 by one-way ANOVA with Dunnett’s post-hoc test (**c**) or Student's *t*-test (**d**). (**e**) Melanin levels in human artificial skin treated with 2'-FL (20 or 40 g/L) for 7 days by spectrophotometric (a; n = 3 per group) and Fontana-Masson staining (b; n = 3 per group) assays. Data are represented as mean ± SD. **P < 0.01 by one-way ANOVA with Dunnett’s post-hoc test. Dotted area in (**f**) shows where pigmentation was reduced. Intensity of control (CTL) samples was used for normalization. Data are represented as mean ± SD. *P < 0.05 by one-way ANOVA with Dunnett’s post-hoc test.

To investigate whether 2'-FL (Fig. 1a) could change the morphology or viability of human melanocytes or MNT-1 cells, the cells were treated with 2'-FL (10 or 20 g/L) for 24 h. Melanocytic cells showed insignificant (*P* > 0.05) changes in cell morphology (Fig. 1b) and viability (Fig. 1c) in the presence of 2'-FL compared with untreated cells, thereby suggesting minimal to no cytotoxicity of 2'-FL. Analysis of mouse B16 cells on day 7 of treatment with 2'-FL (10 or 20 g/L) revealed that the melanin levels were significantly (*P* < 0.05) decreased in both cell lysates and pellets (Fig. 1d). Based on our findings in human melanocyte and MNT-1 cells, and mouse B16 cells, we further examined the inhibitory effects of 2'-FL on hyperpigmentation in the human skin. A human skin model containing human primary melanocytes was treated with 2'-FL (20 or 40 g/L), showing significant (*P* < 0.05) recovery from skin pigmentation by day 7 (Fig. 1e,f). These results indicate that 2'-FL treatment contributes for the reduction of intracellular melanin levels in melanocytic cells and human skin models with minimal cell cytotoxicity and morphological changes.

### Transcriptome analysis reveals 2'-Fucosyllactose is associated with autophagy via the AMPK-ULK axis

**Figure 2:**
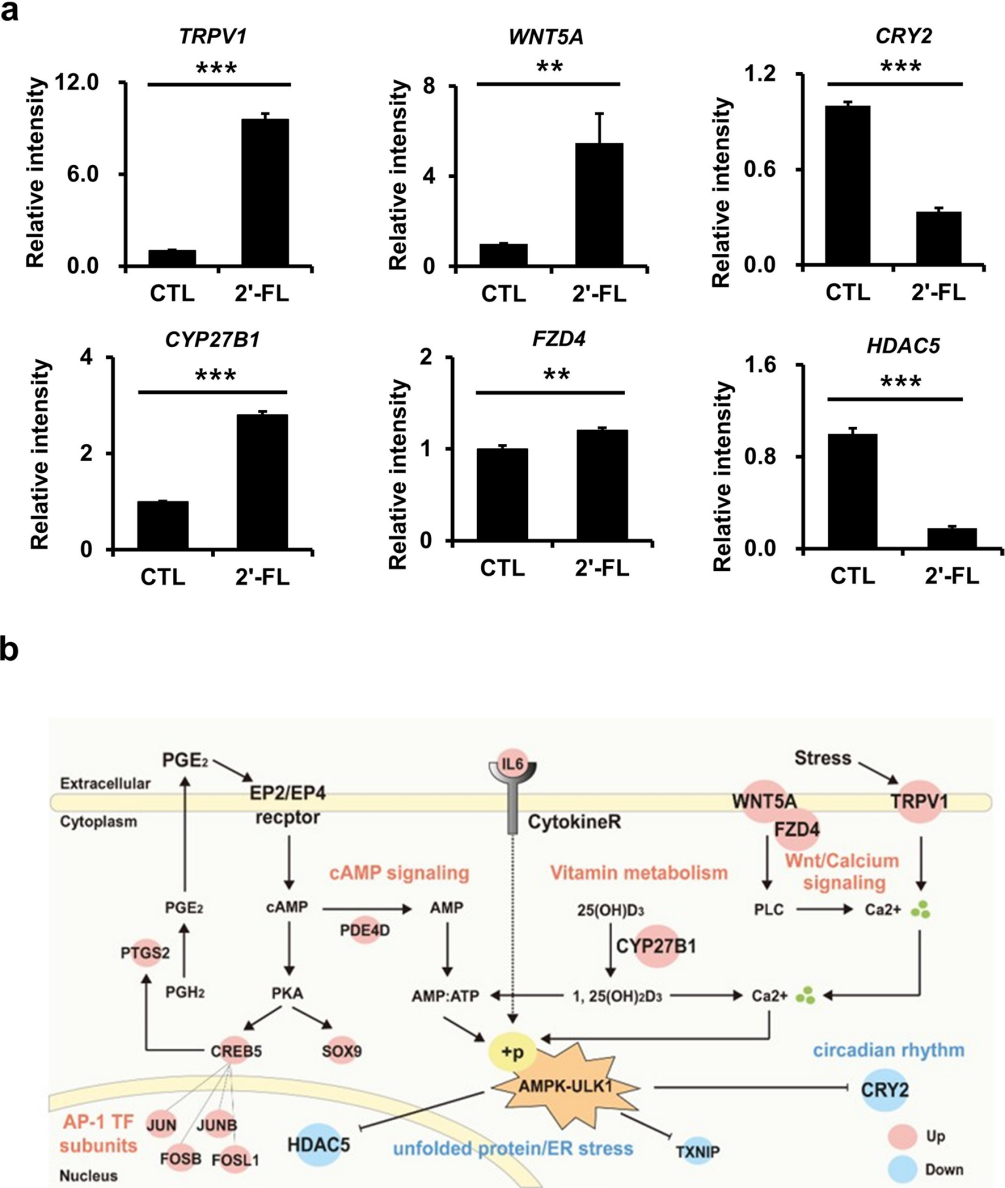
Transcriptome analysis reveals 2'-Fucosyllactose is associated with autophagy via the AMPK-ULK axis. (**a**) Quantitative RT-PCR analysis of six DEGs*. GAPDH* expression was used as reference. Data are represented as mean ± SD. *P < 0.05, **P < 0.01, ***P < 0.001 by Student’s *t*-test. (**b**) Network model describing the relationships between the enriched GOBPs/KEGG pathways upon 2'-FL treatment in. Large nodes indicate the genes examined in (**a**). Gray solid lines denote protein–protein interactions. Arrows and inhibition symbols represent activation and suppression, respectively.

To explore the cellular processes and signaling pathways regulated by 2'-FL treatment, we conducted a systematic transcriptome analysis and subsequent comparison of the gene expression profiles of human MNT-1 cells in the presence or absence of 2'-FL (20 g/L). A total of 1151 differentially expressed genes (DEGs) were identified, comprising 751 upregulated and 400 downregulated genes (Supplementary Fig. S1a and Table S1). Interestingly, none of previously known melanogenic genes, including microphthalmia transcription factor (MITF), were significantly changed (Supplementary Fig. S2), suggesting that the melanin reduction by 2'-FL treatment is due to the activation of melanin degradation, rather than the suppression of melanogenesis. Functional enrichment analysis of Gene Ontology biological processes (GOBPs) and Kyoto Encyclopedia of Genes and Genomes (KEGG) pathways (Supplementary Fig. S1b and Table S2) revealed that the upregulated genes were significantly (*P* < 0.05) involved in Ca^2+^-dependent noncanonical Wnt signaling pathway (cellular response to calcium ion), and responses to vitamin D and cAMP, whereas the downregulated genes were significantly involved in circadian rhythm and endoplasmic reticulum (ER) stress (response to unfolded protein and protein processing in ER). Next, among the DEGs, we further selected genes that represented the aforementioned cellular processes and signaling pathways, or that were associated with the AMPK–ULK1 axis or autophagy for further analyses (Supplementary Fig. S1c), including the upregulated genes representing Ca^2+^-dependent noncanonical Wnt signaling pathway (*TRPV1* ^29,30^, *WNT5A*, *FZD4*, and *EDN1*^31^) and responses to vitamin D (*CYP27B1*) and cAMP (*PDE4D*), the downregulated genes representing circadian rhythm (*CRY2*^32^), and genes associated with the AMPK–ULK1 axis that is directly related to autophagy induction (*TXNIP*^33^, *LIPE*^34^, and *HDAC5*^35,36^). For six of the selected genes, we then confirmed their expression changes in the absence and presence of 2'-FL by quantitative RT-PCR (Fig. 2a). Additionally, to summarize these systematic changes by 2'-FL treatment, we built a molecular network model, describing (i) cAMP (*PDE4D*, *SOX9*, *CREB5*, and *PTGS2*) and Ca^2+^ signaling pathways (*TRPV1*, *WNT5A*, and *FZD4*), vitamin metabolism (*CYP27B1*), and activator protein-1 (AP-1) transcription factor-associated transcriptional regulation that were activated by cAMP and/or calcium signaling pathways (*JUN*, *JUNB*, *FOSB*, and *FOSL1*), and unfolded protein and ER stress (*TXNIP* and *HDAC5*), and circadian rhythm (*CRY2*) as the up- and downstream cellular processes and pathways of the AMPK–ULK1 axis, respectively (Fig. 2b; additional details in Discussion). Therefore, our systematic approach suggested that the AMPK–ULK1 axis functioned as a key signaling pathway in response to 2'-FL treatment, orchestrating its up- and downstream cellular and molecular mechanisms.

### 2'-Fucosyllactose induces autophagy via the phosphorylation of AMPK and ULK1

Previous studies have suggested autophagy as a critical regulator of melanosomes in keratinocytes, as well as for determining human skin color^6,8^. In particular, the AMPK–ULK1 axis, in which the activated AMPK harboring Thr172 phosphorylation (p-AMPK) directly phosphorylates ULK1 (Ser555), is known to induce autophagic melanin degradation^19,20,37^.

**Figure 3:**
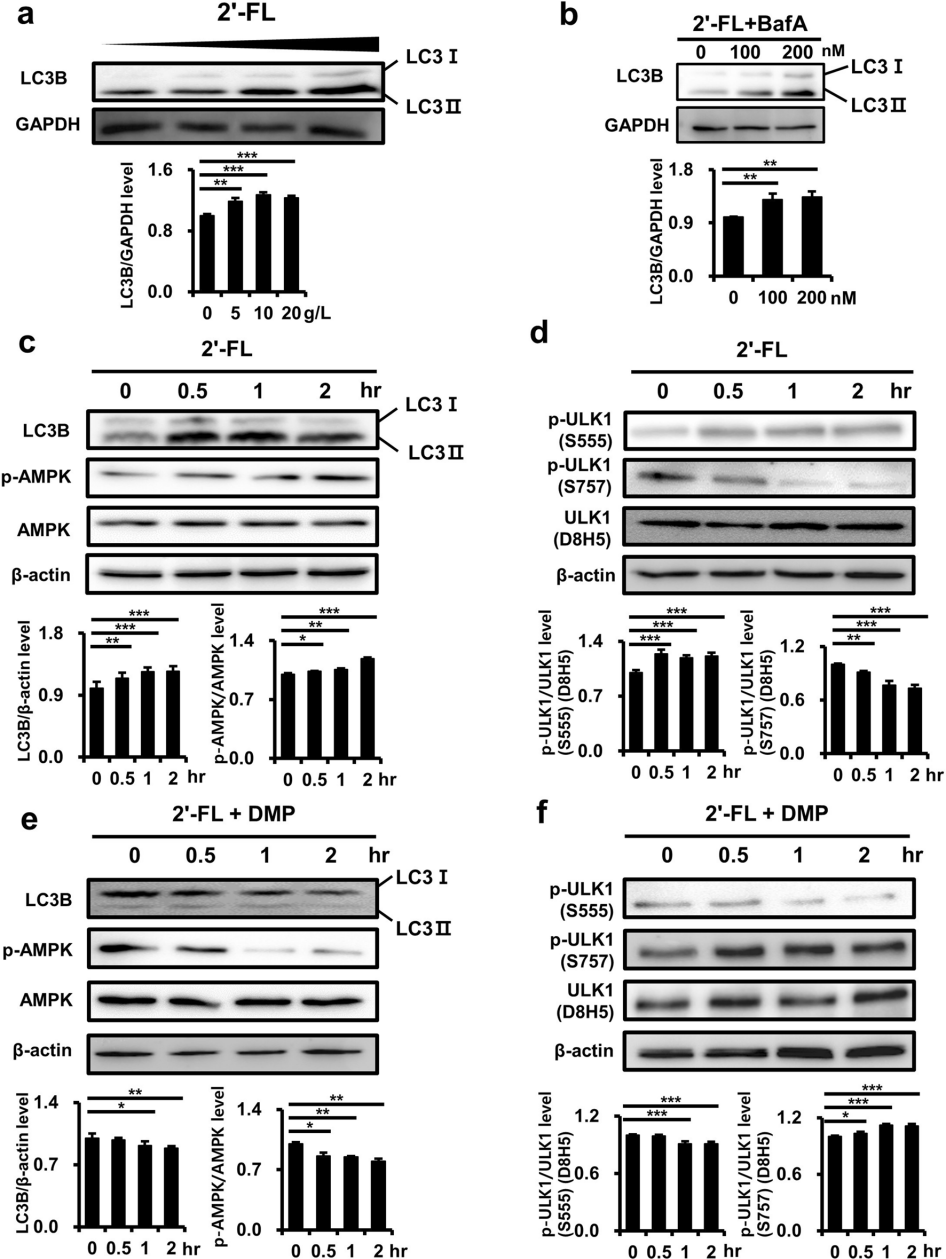
2'-Fucosyllactose induces autophagy via the phosphorylation of AMPK and ULK1. (**a**) Western blot analysis of LC3II levels in human MNT-1 cells treated with 2'-FL (0, 5, 10 or 20 g/L) for 2 h. (**b**) Western blot analysis of LC3 protein levels in MNT-1 cells treated with 2'-FL (20 g/L) and Baf A (0, 100, and 200 nM) for 2 h. (**a**, **b**) The graph indicates the relative protein levels of LC3B normalized by GAPDH. (**c**–**f**) Western blot analysis of LC3II, total AMPK, and phosphorylated AMPK (Thr172) (**c**, **e**) as well as of total and phosphorylated (Ser555 and Ser757) ULK1 (**d**, **f**) in human MNT-1 melanoma cells treated with 2'-FL (20 g/L) alone (**c**, **d**) or plus dorsomorphin (10 µM, DMP) (**e**, **f**) for 0, 0.5, 1, and 2 h. (**c**, **e**) Phosphorylation level of LC3B normalized by β-actin and phosphorylation level of AMPK normalized by AMPK. (**d**, **f**) Phosphorylation level of ULK1 normalized by ULK1, *P < 0.05, **P < 0.01, ***P < 0.001 by Student’s *t*-test.” (Also see Supplementary Fig. 2). LC3, microtubule-associated protein 1A/1B-light chain 3; Baf A, Bafilomycin A; AMPK, adenosine monophosphate activated protein kinase; ULK1, Unc-51-like autophagy activating kinase 1.

2'-FL treatment induced microtubule-associated protein 1A/1B-light chain 3 (LC3)I conversion into LC3II, an indication of autophagy activation, in MNT-1 cells in a dose dependent manner (Fig. 3a). Bafilomycin A (Baf A), an inhibitor of autophagosome-lysosome fusion, readily increased LC3II level (Fig. 3b), indicating increased autophagic flux by 2'-FL treatment. Moreover, accumulation of LC3II and p-AMPK (Thr172) were found to increase after 2'-FL treatment, whereas AMPK levels were not significantly changed (Fig. 3c). Furthermore, p-ULK1 at Ser555 was induced at 0.5 h and reached a plateau upon 1–2 h of treatment, whereas phosphorylation at Ser757 started to reduce and total ULK1 levels remained unaltered (Fig. 3d). Considering the temporal correlations between accumulation of LC3II and phosphorylation of AMPK and ULK1, it is plausible that 2'-FL may activate autophagy via the AMPK–ULK1 signaling axis.

To test this hypothesis, the activation of autophagy under 2'-FL treatment was investigated next in the presence of dorsomorphin (DMP), a specific AMPK inhibitor that blocks AMPK phosphorylation. As expected, DMP blocked p-AMPK (Thr172) (Fig. 3e) and p-ULK1 (Ser555), whereas it enhanced p-ULK1 at Ser757 (Fig. 3f). Intriguingly, DMP effectively reduced 2'-FL-induced LC3II accumulation, indicating the inhibition of autophagy activation (Fig. 3d). Thus, these findings suggest that 2'-FL activates autophagy via the AMPK–ULK1 axis, thereby inducing hypopigmentation.

### Dorsomorphin treatment blocks 2'-Fucosyllactose-induced autophagosome formation

**Figure 4:**
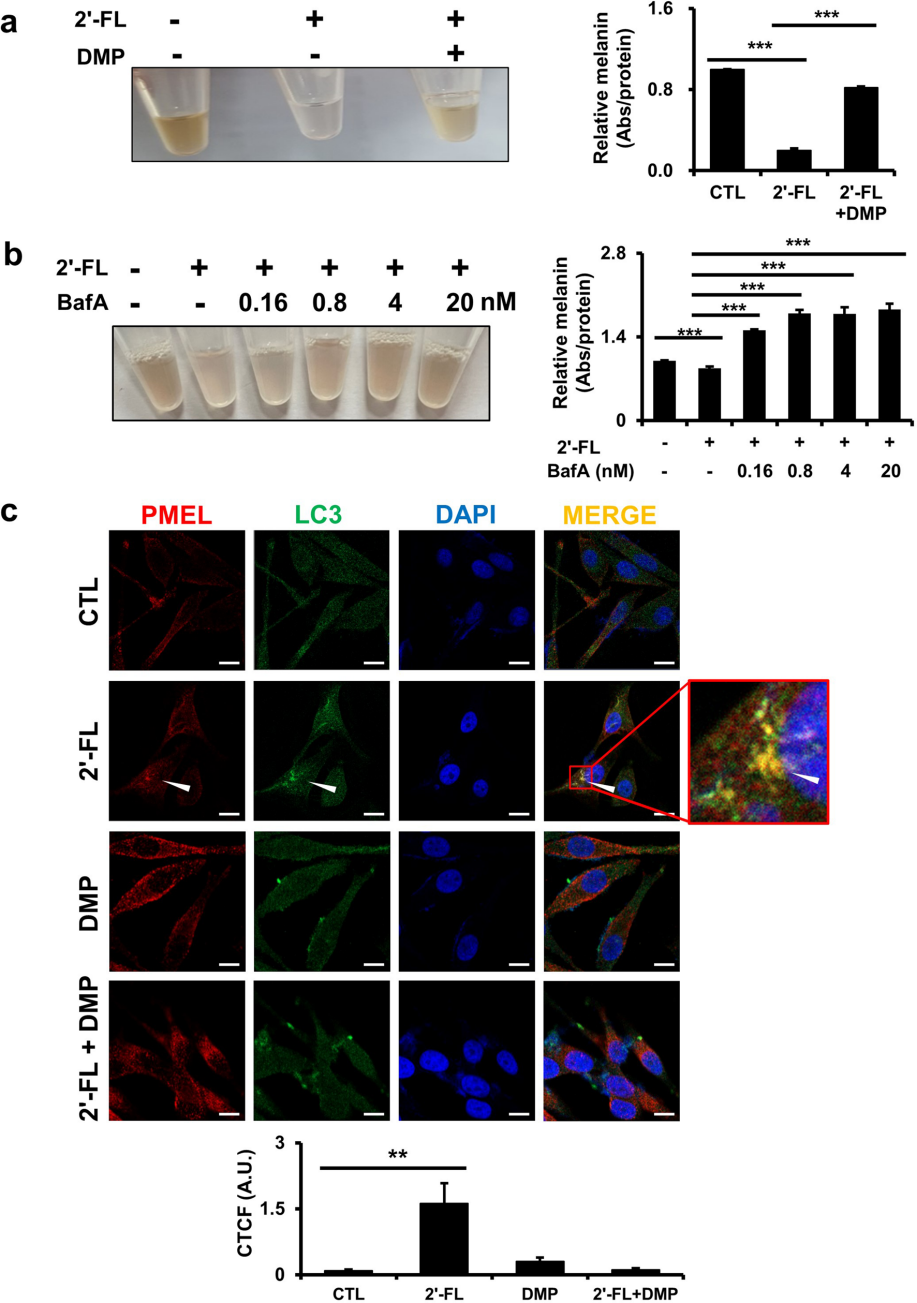
Dorsomorphin treatment blocks 2'-Fucosyllactose-induced autophagosome formation. (**a**) Melanin levels in human MNT-1 cells treated for 7 days with 2'-FL (20 g/L) alone or combined with DMP (10 µM). (**b**) Melanin levels in human MNT-1 cells treated for 5 days with 2'-FL (20 g/L) alone or in combination with Baf A (0.16, 0.8, 4, and 20 nM). (**c**) Immunohistochemical analysis (magnification: × 400) of PMEL (green) and LC3 (red) in human MNT-1 cells treated with 20 g/L 2'-FL and/or 10 μM DMP for 24 h. Cell nuclei are stained with DAPI (blue). Arrows denote colocalization of PMEL and LC3. Fluorescence intensity from cells was quantified using the corrected total cell fluorescence formula (CTCF). mean ± SD. *P < 0.05, **P < 0.01, ***P < 0.001 by Student’s *t*-test. Scale bar = 10 µm. PMEL, premelanosome protein; A.U., arbitrary unit.

Given that 2'-FL was found to activate autophagy via the AMPK–ULK1 axis, we first compared the melanin levels in human MNT-1 cells treated with 2'-FL (20 g/L) in the presence or absence of DMP, confirming that the inhibition of the AMPK–ULK1 axis prevented 2'-FL-mediated hypopigmentation (Fig. 4a). Autophagy inhibitors readily recovered melanin production in 2'-FL treated cells (Fig. 4b, Supplementary Fig. S4). Additional immunofluorescence analysis showed that 2'-FL induced the clearance of melanosomes by fusion with autophagosomes, as denoted by the double-positive signal of the melanosomal marker premelanosome protein (PMEL) and the autophagosomal marker LC3 (2'-FL panel in Fig. 4b). In contrast, when both 2'-FL and DMP were present, LC3B puncta signal was weak and no PMEL/LC3B signals were detected (2'-FL + DMP panel in Fig. 4c). Collectively, these data suggest that 2'-FL-mediated activation of the AMPK–ULK1 axis induces the formation of autophagosomes, leading to melanin degradation.

### Model of 2'-Fucosyllactose-induced autophagy pathway

**Figure 5:**
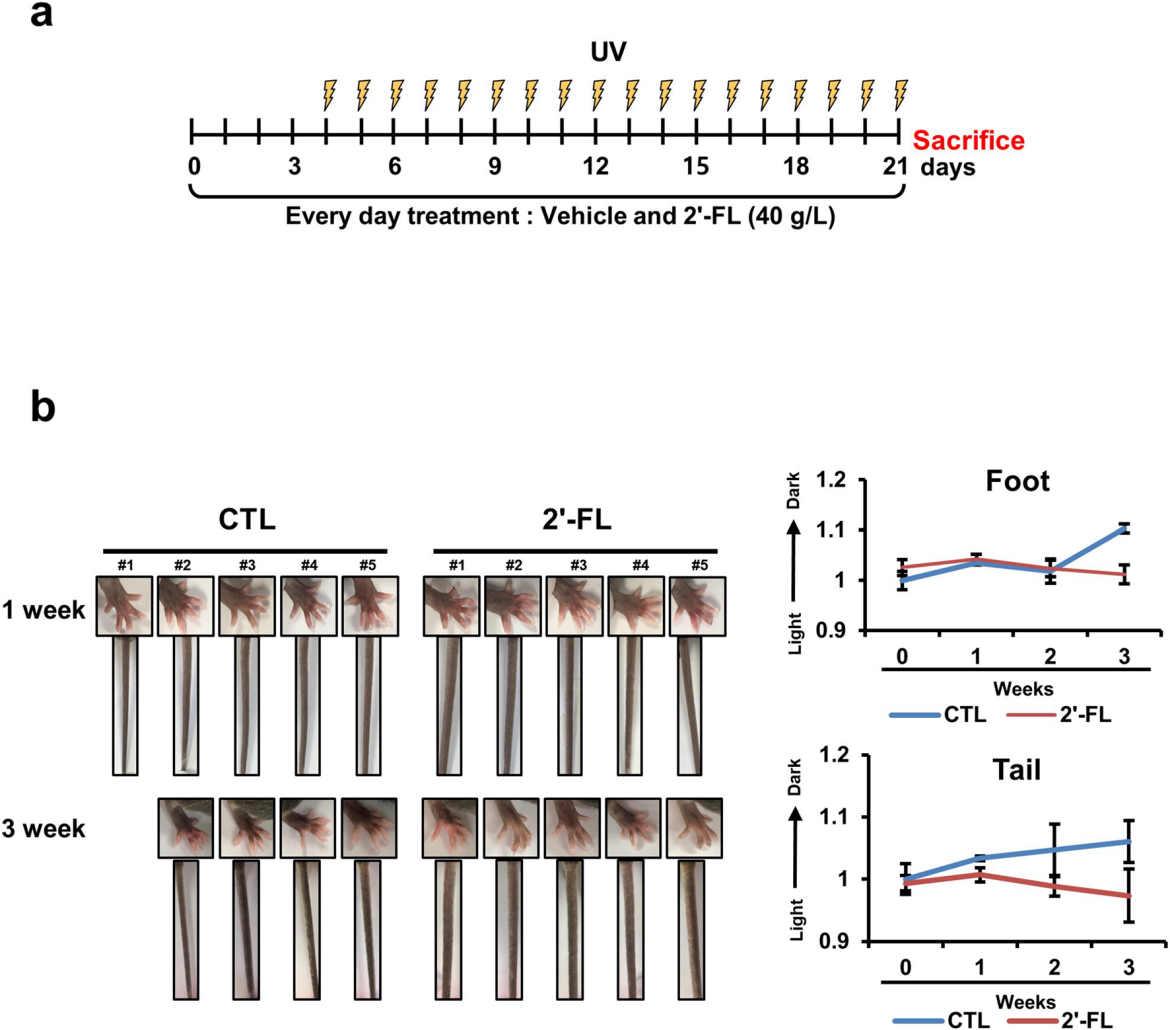
2'-Fucosyllactose reduces melanin production in a mice model. (**a**) Schedule of the in vivo experiment. (**b**) Feet and tail pigmentation differences between control (n = 4) and 2'-FL-treated groups were measured by color analysis. L-values before treatment were used as reference. Low and high relative L-value indicate darkness and whiteness, respectively.

We tested the melanin-degrading effects of 2'-FL in an in vivo mouse model with local application of 2'-FL on the mouse feet and tail together with UVB exposure (Fig. 5a). UVB exposure led to an increase in pigmentation on the foot and tail of the mice without any treatment, whereas this outcome was attenuated in 2'-FL-treated mice (Fig. 5b), suggesting a therapeutic potential of 2'-FL against hyperpigmentation. Moreover, 2'-FL was confirmed to also mediate LC3I conversion into LC3II in vivo (Supplementary Fig. S2).

## Discussion

**Figure 6:**
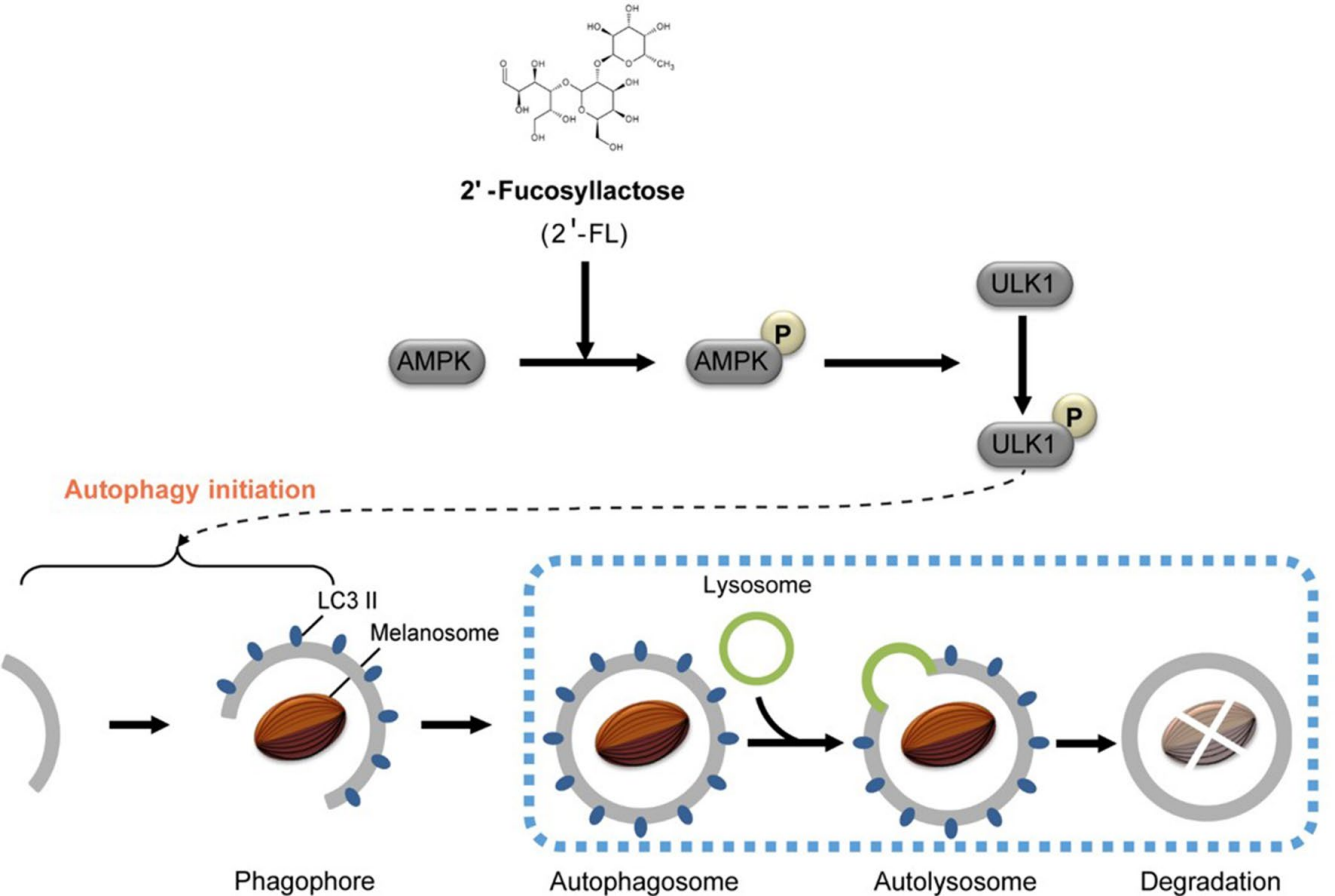
Model of 2'-FL-induced autophagy pathway. 2'-FL activates AMPK, which in turn phosphorylates ULK1 at Ser555 and inhibits Ser757-mediated inactivation, with consequent autophagy initiation. This mechanism results in the degradation of melanin via the formation of autophagosomes.

The present study shows that 2'-FL reduces melanin levels in B16 murine cells, in a human skin equivalent in vitro model, and in a mouse model. Melanin reduction is induced by the clearance of melanosomes through autophagy, which is activated by the AMPK–ULK1 axis. Immunofluorescence analysis confirmed that 2'-FL promotes the formation of autophagosomes. Moreover, transcriptome analysis revealed that the cellular processes and signaling pathways regulated by 2'-FL are also involved in the AMPK–ULK1 axis. Hence, these data reveal previously unknown functions of 2'-FL, including regulation of autophagy through the AMPK–ULK1 axis and melanin degradation (Fig. 6).

Commonly, autophagy can be regulated by the activation of the AMPK–ULK1 axis^19,20,37^. Our results also showed that this signaling axis is a major component of 2'-FL-induced autophagy; however, it is not clear how 2'-FL activates AMPK in human melanocytic cells. The activation of AMPK by Thr172 phosphorylation can be regulated by a perturbation in the cAMP level^38^, and an increase of intracellular Ca^2+^ ^39,40^ and vitamins^41,42^. According to our transcriptome data, Ca^2+^-dependent noncanonical Wnt signaling pathway and response to cAMP were all upregulated by 2'-FL treatment, suggesting the activation of upstream pathways of the AMPK–ULK1 axis. Of note, previous studies showed that those cellular pathways are also involved in the induction of autophagy^43,44^. Intriguingly, other cellular processes and signaling pathways identified as being affected by 2'-FL were also reported to be associated with the AMPK signaling pathway. ER stress and circadian rhythm, which were represented by 2'-FL-induced downregulated genes, are also associated with AMPK signals and/or autophagy^45^. Furthermore, AP-1 transcription factor complex, which is known to be regulated by cAMP and/or calcium signaling^46^, is also regulated by the AMPK–ULK1 axis^47^. However, concerning melanin degradation by 2'-FL treatment, the detailed relationships of the AMPK–ULK1 axis and autophagy with those cellular processes and signaling pathways remain to be elucidated.

Assuming the negligible extracellular transport of melanin, which could be speculated by the similar patterns of melanin reduction in both cell lysates and pellets in our study (Fig. 1d), intracellular melanin level is simply determined by the balance of melanin production and degradation processes. Although the total amount of melanin can be measured by diverse experimental methods, the melanin production to degradation ratio has barely been measured due to the lack of indicative markers for melanin degradation. We demonstrated that colocalization of LC3 and PMEL is an indicative marker of melanosome degradation in autophagosomes. Although previous studies^48,49^ demonstrated that LC3 can participate in melanogenesis by upregulating MITF, our study showed that expression levels of the melanogenic genes were not significantly changed (Supplementary Fig. S1), suggesting that LC3-I conversion to LC3-II contributes to melanin degradation via autophagy, but not to melanogenesis. Thus, Temporal changes in LC3^+^/PMEL^+^ marker may afford a new strategy to evaluate the degradation rate of melanin, thereby enabling to clarify the reduction of melanin levels as either suppression of melanogenesis or acceleration of melanin degradation. This detailed clarification can be further applied to better understand the underlying mechanisms of antimelanogenic or melanin-degrading agents. Nevertheless, further investigations are necessary to identify the specific molecular and functional characteristics of melanosome-specific autophagosomes, which are distinct from both conventional lysosomes and melanosomes.

In summary, this study demonstrates that human milk oligosaccharide-originated 2'-FL reduces melanin levels in human MNT-1 cells and skin equivalents, with autophagy via activation of the AMPK–ULK1 axis as the key mechanism mediating melanin degradation. Thus, we propose 2'-FL as a potential melanin-degrading agent with high stability and good safety profile. Moreover, we believe that our approach provides new insights into the identification of potential antimelanogenic or melanin-degrading agents, as well as their related key mechanisms.

## Materials and methods

### Cell culture and materials

The MNT-1 cell line (human melanoma) was kindly provided by Dr. Ai-Young Lee at Dongguk University, who originally received it as a gift from Dr. Vincent J. Hearing from the National Institutes of Health. The cells were cultured at 37 °C in EMEM (Gibco, Waltham, MA, USA) with 20% fetal bovine serum (FBS; GenDepot, Katy, TX, USA), 10% high-glucose DMEM (Lonza, Basel, Switzerland) as high energy source, 20 mM HEPES, and penicillin–streptomycin (Welgene, Gyeongsan, Republic of Korea). Normal human melanocytes were purchased from the Korean Cell Line Bank and were maintained at 37 °C in Medium 254 (Cascade Biologics, Waltham, MA, USA) with human melanocyte growth supplement (Life Technologies, Waltham, MA, USA) and penicillin–streptomycin. 2'-FL and DMP were obtained from Sigma-Aldrich (St. Louis, MO, USA). The B16 cell line (mouse melanoma) was purchased from the ATCC (Manassas, VA, USA) and was cultured in DMEM containing 10% FBS and 1% penicillin–streptomycin at 37 °C in a 5% CO_2_ atmosphere.

### Cell viability assay

Cell viability was assessed by the MTT assay. Briefly, 1.5 × 10^5^ cells were seeded in 6-well plates, incubated for 24 h, and then treated with 2'-FL (10 or 20 g/L) for 24 h. The medium was then replaced by 1 mL of MTT solution (Sigma-Aldrich) for 4 h. The cells were collected, washed, and resuspended in 800 µL of DMSO. The optical density (absorbance) was measured at 570 nm using SpectraMax 190 Absorbance Plate Reader (Molecular Devices, San Jose, CA, USA).

### Melanin assay

B16 cells were treated with RIPA buffer, and the pellets and supernatants were collected by centrifugation at 16,600 × g for 15 min. The supernatants were subjected to protein quantification analysis by BCA protein assay (Thermo Fisher Scientific, Waltham, MA, USA). The pellets containing melanin were dissolved in 1 M NaOH and incubated for 1 h at 80 °C. Both protein and melanin contents were spectrophotometrically determined by measuring the absorbance at 562 and 450 nm, respectively.

### Quantitative RT-PCR

Total RNA was extracted with the TRIzol RNA Reagent (Life Technologies) according to the manufacturer’s instructions. After resuspension of the extracted RNA in 50 μL of nuclease-free water, its concentration was determined using a BioPhotometer (Eppendorf, Hamburg, Germany). The extracted RNA samples were stored at − 80 °C until use. cDNA was reverse transcribed from an equal amount of RNA using Moloney Murine Leukemia Virus Reverse Transcriptase (75,142; Qiagen, Hilden, Germany) with oligo (dT) as primer. Quantitative RT-PCR was performed using CYBR Q GreenBlue qPCR Master Mix (CellSafe, Yongin, Republic of Korea) and the AriaMx Real-Time PCR System (Agilent Technologies, Santa Clara, CA, USA).

mRNA expression was measured using specific primers: *TRPV1* forward (5'–ATCGCAGGAGTATCTTTGAA–3') and reverse (5'–TGTCTCAGGGTCTTTGAACT–3'); *WNT5A* forward (5'–CAGGACTTTCTCAAGGACAG–3') and reverse (5'–AGAGGTGTTATCCACAGTGC–3'); *FZD4* forward (5'–AGACTGATGGTCAAGATTGG–3') and reverse primer (5'–CCCAGTTGGAGATTTCATAA–3'); *CYP27B1* forward (5'–ACAGCACTCCACTCAGAGAT–3') and reverse (5'–CTTAGCACTTCCTTGACCAC–3'); *HDAC5* forward (5'–TACAGCAGAAGTTGAACGTG–3') and reverse (5'–ATAGCGATGCAGAGAGATGT–3'); *CRY2* forward (5'–TATGACTCTGAACCCTTTGG–3') and reverse (5'–GATCCTGTCCAGGTCATAGA–3') primers.

### Western blot analysis

The cells were lysed with 1% NP-40 in a solution of 0.05 M Tris–HCl (pH 7.5), 0.15 M NaCl, 0.01 M MgCl2, and a protease inhibitor cocktail (P8340; Sigma-Aldrich). After centrifuging the samples at 16,600 × g for 15 min, the supernatant was collected and its protein content was quantified by BCA protein assay (Thermo Fisher Scientific). More than 20 µg of total protein was electrophoretically separated in a SDS–polyacrylamide gel and transferred onto polyvinylidene fluoride membranes, which were incubated for 24 h at 4 °C with each of the following antibodies (1:4,000 dilution): anti-GAPDH, anti-LCB, anti-AMPK, anti-p-AMPK, anti-β-actin, anti-p-ULK1 (Ser555), anti-phosphoULK1 (Ser757), or anti-ULK1 (D8H5) (Cell Signaling Technology, Danvers, MA, USA). Then, the membranes were washed and incubated with horseradish peroxidase-conjugated secondary antibody (GenDEPOT) at a concentration of 1:10,000 for 1 h at 25 °C. The proteins were detected using enhanced chemiluminescence reagent (Thermo Fisher Scientific), and images were captured using an FUSLON SOLOS (Korea Biomics, Seoul, Republic of Korea). The band intensities were analyzed using ImageJ software (NIH, USA). The relative protein levels of LC3B were normalized to GAPDH, phospho-AMPK was normalized to AMPK, and ULK1 phosphorylation was normalized using ULK1.

### Immunofluorescence

Cells were cultured on Lab-Tek chamber slides (Thermo Fisher Scientific), fixed with 4% paraformaldehyde in phosphate-buffered saline (PBS), and permeabilized with 0.1% Triton X-100 in PBS containing 1% BSA for 5 min. Afterwards, the cells were incubated with anti-PMEL (Invitrogen, Waltham, MA, USA), anti-LCB antibody (Cell Signaling Technology), and DAPI (Molecular Probes, Eugene, OR, USA). Fluorescence was detected by secondary antibody staining with Alexa Fluor 488-conjugated F(ab’)2 fragment of goat anti-mouse IgG and Alexa Fluor 594-conjugated F(ab’)2 fragment of goat anti-rabbit IgG (Invitrogen). All images were acquired using an LSM 800 confocal microscope (Zeiss, Jena, Germany). The fluorescence intensity of PMEL and LC3 co-expression was quantified using ImageJ, and displayed in corrected total cell fluorescence (CTCF). Graphs depict the quantification of cell integrated density among CTL, 2'-FL, DMP, and 2'-FL + DMP in the four experimental groups. To measure intensity, total cell fluorescence (CTCF) was calculated using the formula: CTCF = Integrated Density − (Area of selected cell × Mean fluorescence of background readings).

### In vitro 3D skin model

KeraSkin-M™ (Biosolution, Seoul, Korea), which is a model that is reconstructed similarly to the skin using primary human keratinocytes and melanocytes^50^, was used. The melanocytes present in the basal cell layer derived from dendritic cells and produced melanin granules. When cultured in an air liquid interface culture for 2 weeks, it gradually became pigmented, formed multilayers, and fully differentiated.

### Animals

All animal experiments were approved by the Institutional Animal Care and Use Committee (IACUC) of Semyung University (Approval no. smecae 20-04-01), and were performed according to the animal testing guidelines. Experiments were carried out in accordance with the animal protocols approved by the IACUC at the University of Semyung. All the animal experiments followed the ARRIVE guidelines.

### In vivo skin color analysis

Twelve-week-old male C57BL6 mice were randomly divided into four control and five experimental groups. For the first 4 days, the experiment was conducted without UVB irradiation to determine 2'-FL-promoted skin irritation. Afterwards, the animals were allowed to rest for 2 days, and were then exposed for 5 days to UVB irradiation and 2'-FL (40 g/L diluted in water) treatment. After irradiation with UVB light (302 nm, KUT-0820M; Korea Lab Tech, Namyangju, Republic of Korea) for 10 min, the control group remained untreated, whereas and the experimental group was applied 100 μL of 2'-FL to the foot and tail. The foot and tail were photographed weekly, and the skin color was measured. Skin color analysis was performed using a General Colorimeter (JZ-300, Kingwell Instrument Co., Shenzhen, China). The measured data scale was from 0 to 100, with low L-value indicating darkness, and high L-value indicating whiteness. All mice were subjected to triplicate measurement, color analysis was performed, and a value was calculated as an average.

### Statistical analysis

Differences between three groups were assessed by analysis of variance (ANOVA) with Dunnett’s post-hoc test and between two groups were determined by Student’s t-test. *P*-values < 0.1 and < 0.05 were considered statistically significant for quantitative RT-PCR assays in Fig. 2d and the other experiments, respectively.

## Supplementary Information

Supplementary Information.

## Data Availability

The raw data for RNA sequencing was deposited in Korean Nucleotide Archive (KoNA, https://kobic.re.kr/kona) under accession number PRJKA220163.
