# Author Correction: Human milk oligosaccharide 2'-fucosyllactose promotes melanin degradation via the autophagic AMPK–ULK1 signaling axis

**DOI:** 10.1038/s41598-022-20541-9

**Published:** 2022-09-20

**Authors:** Hyojin Heo, Byungsun Cha, Dongmin Jang, Chaewon Park, Gunwoo Park, Byeong-Mun Kwak, Bum-Ho Bin, Ji-Hwan Park, Mi-Gi Lee

**Affiliations:** 1 Department of Applied Biotechnology, Ajou University, Suwon, 16499 Republic of Korea; 2 Department of Biological Sciences, Ajou University, 206 Worldcup-ro, Yeongtong-gu, Suwon, 16499 Republic of Korea; 3 Department of Biomedical Sciences, Ajou University Graduate School of Medicine, Suwon, 16499 Republic of Korea; 4 Korea Bioinformation Center, Korea Research Institute of Bioscience and Biotechnology, Daejeon, 34141 Republic of Korea; 5 School of Cosmetic Science and Beauty Biotechnology, Semyung University, Chungbuk, 27136 Republic of Korea; 6 Gyeonggido Business and Science Accelerator, 107 Gwanggyo-ro, Yeongtong-gu, Suwon, 16229 Republic of Korea

Correction to: *Scientific Reports* 10.1038/s41598-022-17896-4, published online 17 August 2022

In the original version of this Article Dongmin Jang was omitted from the equally contributing authors statement.

“These authors contributed equally: Hyojin Heo and Byungsun Cha”.

now reads:

“These authors contributed equally: Hyojin Heo, Byungsun Cha and Dongmin Jang”.

The original Article has been corrected.

