# Supplementary material for: Human milk oligosaccharide 2'-fucosyllactose promotes melanin degradation via the autophagic AMPK–ULK1 signaling axis
Source: Sci Rep. 2022 Aug 17;12:13983. doi: 10.1038/s41598-022-17896-4 (PMC9385628; doi:10.1038/s41598-022-17896-4)
Supplement: Supplementary file 1 — Supplementary Information. [file 41598_2022_17896_MOESM1_ESM.docx]

*Supplementary Information*

**Human milk oligosaccharide** **2'**-**fucosyllactose promotes melanin degradation via the autophagic AMPK–ULK1 signaling axis**

Hyojin Heo^1, #^, Byungsun Cha^2, #^, Dongmin Jang^3, 4^, Chaewon Park^1^, Geon-woo Park^1, 4^, Byeong-Mun Kwak^5^, Bum-Ho Bin^1, 2, *^, Ji-Hwan Park^4, *^, Mi-Gi Lee^6, *^

^#^These authors contributed equally to this paper.

To whom correspondence should be addressed: [bhb@ajou.ac.kr](about:blank) (Bum-Ho Bin), or [jhpark706@kribb.re.kr](about:blank) (Ji-Hwan Park), or [migi@gbsa.or.kr](about:blank) (Mi-Gi Lee)

**This file includes:**

1. Supplementary Materials and Methods;
2. Supplementary Tables S1 and S2
3. Supplementary Figure S1; and
4. Supplementary Figure S2; and
5. Supplementary Figure S3; and
6. Supplementary Figure S4; and
7. Supplementary References;

**Supplementary Materials & Methods**

**RNA isolation and sequencing analysis**

Purity of the extracted RNA was determined using the NanoDrop 8000 spectrophotometer (Thermo Fisher Scientific, Waltham, MA, USA). RNA integrity number was measured by 2100 BioAnalyzer (Agilent Technologies, Waltham, MA, USA) and it was confirmed to be higher than seven in all samples. Poly(A) mRNA was isolated and fragmented from total RNA (1 µg) using the TruSeq Stranded mRNA Library Prep Kit (Illumina, San Diego, CA, USA) and poly-T oligo-attached magnetic beads. Following the reverse transcription of the fragmented mRNA, strand-specific cDNA libraries were constructed with adaptor-ligation reactions and the quality of the amplified cDNA was confirmed by capillary electrophoresis with the 2100 BioAnalyzer. The libraries were then sequenced using Illumina HiSeq 2500 in paired-end mode (2 × 100 bp).

To analyze the RNA sequencing data it was used the FastQC software (v.0.11.5, https://www.bioinformatics.babraham.ac.uk/projects/fastqc/) to assess the quality of the raw read sequences and Cutadapt v.2.7 algorithm[^1^](#_ENREF_1) to trim the adapter sequences in paired-end reads (for read-1, TruSeq index adapter with the option “-a AGATCGGAAGAGCACACGTCTGAACTCCAGTCAC”; and for read-2, the reverse complementary sequence of TruSeq universal adapter with the option “-a AGATCGGAAGAGCGTCGTGTAGGGAAAGAGTGTAGATCTCGGTGGTCGCCGTATCATT”). Then the sequences were aligned based on the GRCh38 (release 100) genome[^2^](#_ENREF_2) using STAR v.2.6.0c algorithm[^3^](#_ENREF_3), with the option to allow the number of mismatches ≤2 or 3% of read length. The MarkDuplicates of Picard Toolkit v.1.84 (http://broadinstitute.github.io/picard/) was used to remove amplification duplicates, and HTSeq-count v.0.11.1[^4^](#_ENREF_4) was used to compute the number of reads (read counts) aligned onto each annotated gene in GRCh38.

**Identification of differentially expressed genes (DEGs)**

The trimmed mean of M-values (TMM) normalization based on the read counts was performed using edgeR v.3.20.9[^5^](#_ENREF_5) to obtain counts per million mapped reads (CPM) values. One pseudo-value was added to the CPM values of each individual sample, followed by log_2_-transformation. Quantile normalization was then applied to the CPM values[^6^](#_ENREF_6) and the genes with their log_2_-CPM >1 in at least one sample were determined as the expressed genes[^7^](#_ENREF_7). To identify DEGs between melanocyte samples in the absence or presence of 2'-fucosyllactose (2'-FL), the integrative statistical test was applied as previously described[^8^](#_ENREF_8). Briefly, Student’s *t*-test and log_2_-median-ratio test were applied to the log_2_-CPM values of each gene, obtaining *T*-value and log_2_-median-ratio. Next, empirical null distributions for *T*-values and log_2_-median-ratios were generated by applying random sampling experiments 1,000 times. Two-tailed test was used to compute the adjusted *P*-values for the observed *T*-value and log_2_-median ratio of each gene, which were then combined to calculate an overall *P*-value by using Stouffer’s method[^9^](#_ENREF_9). Genes were deemed as DEGs when the overall *P*<0.05 and absolute log_2_-median-ratios >0.4365, which represented the mean of 5^th^ and 95^th^ percentiles of the empirical null distribution for log_2_-median-ratio.

**Functional enrichment analysis**

To identify the cellular processes and signaling pathways represented by the DEGs, functional enrichment analysis of Gene Ontology biological processes (GOBPs)[^10^](#_ENREF_10) and Kyoto Encyclopedia of Genes and Genomes (KEGG) pathways[^11^](#_ENREF_11) was performed using DAVID software[^12^](#_ENREF_12). GOBPs and KEGGs represented by the DEGs were identified as the ones with the *P*-values computed from DAVID <0.05 and the counts >3.

**Construction of a molecular network model**

To build a network model for the representative cellular processes and signaling pathways significantly (*P*<0.05) enriched by the up- and downregulated genes, we first selected a subset of the identified DEGs that are annotated in the GOBP/KEGG pathways in Figure 4c. Using Cytoscape software[^13^](#_ENREF_13), we visualized protein-protein interactions among the selected genes based on 448,486 interactions for 18,169 protein collected from the following five interactome databases: the Biological General Repository for Interaction Datasets (BioGRID)[^14^](#_ENREF_14), the IntAct molecular interaction database (IntAct)[^15^](#_ENREF_15), the Molecular INTeraction database (MINT)[^16^](#_ENREF_16), the Database of Interacting Proteins (DIP)[^17^](#_ENREF_17), and the Interologous Interaction Database (I2D)[^18^](#_ENREF_18). The nodes of the resulting network were further arranged by using the information in the KEGG pathway database or literature (for further details see Discussion).


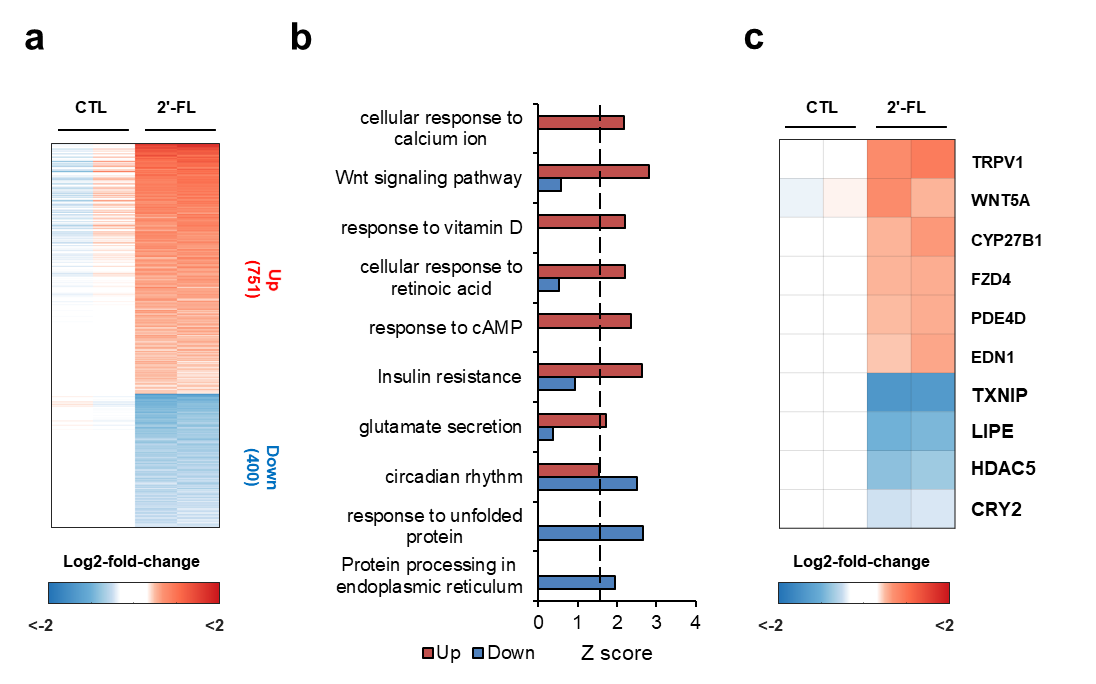


**Supplementary Figure S1. 2'-FL is related to autophagy via the AMPK-ULK axis, according to transcriptome analysis.**

(a) Up- (red) and downregulated (blue) genes in human MNT-1 cells upon 2 h treatment with 2'-FL (20 g/L) compared with nontreated cells (control). Number of differentially expressed genes (DEGs) is denoted in parentheses. (b) Gene Ontology biological processes (GOBPs) and Kyoto Encyclopedia of Genes and Genomes (KEGG) pathways enriched by the up- (red) or downregulated (blue) genes. Bars represent the *Z*-scores converted from the enrichment *P*-values of the corresponding GOBPs/KEGG pathways, as defined by *Z*=*N*^−1^(1−*P*), where *N*^−1^(...) represent the inverse standard normal distribution. Dashed black line denotes the threshold for enrichment significance (*P*<0.05). (c) Expression of genes selected among the DEGs related to the GOBPs/KEGG pathways in (b), or the AMPK–ULK1 axis and autophagy.

**
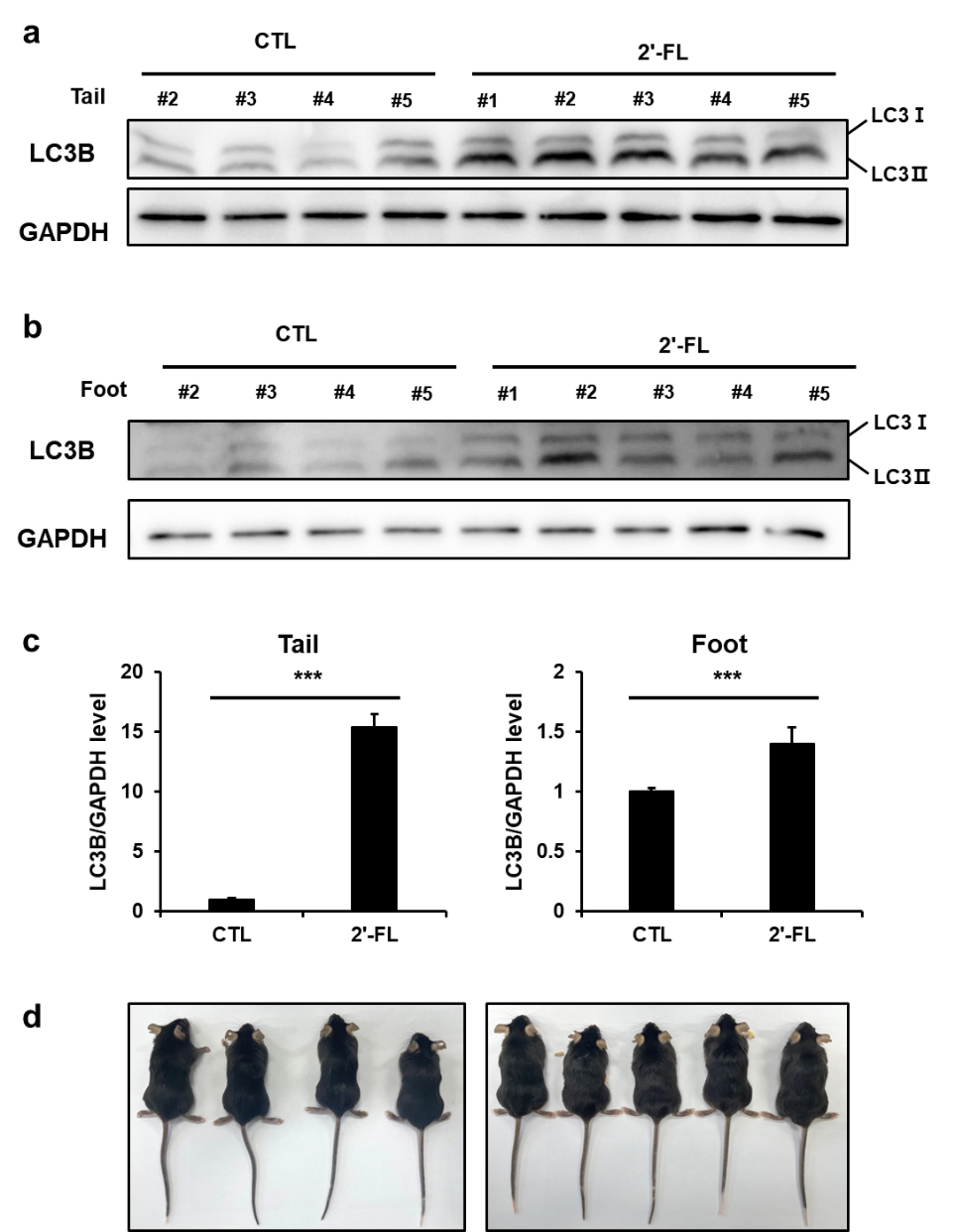
**

**Supplementary Figure S2. 2'-FL treatment induces autophagy in the foot and tail of hyperpigmented mice by exposure to UVB.**

After mice were exposed to UVB with or without 2'-FL treatment (according to the schedule described in Fig. 5c), their foot and tail tissues were subjected to western blot analysis using anti-LC3 and anti-GAPDH antibodies. For the tail (a) and foot tissues (b), we compared the levels of LC3II between 2'-FL-treated (n = 5) and control mice (n = 4). GAPDH was used as internal control. Quantification of LC3B normalized to GAPDH as shown in (c). Photographs of mice corresponding to the individual lanes are shown together (d). CTL, Control; 2'-FL, 2'-fucosyllactose.

**
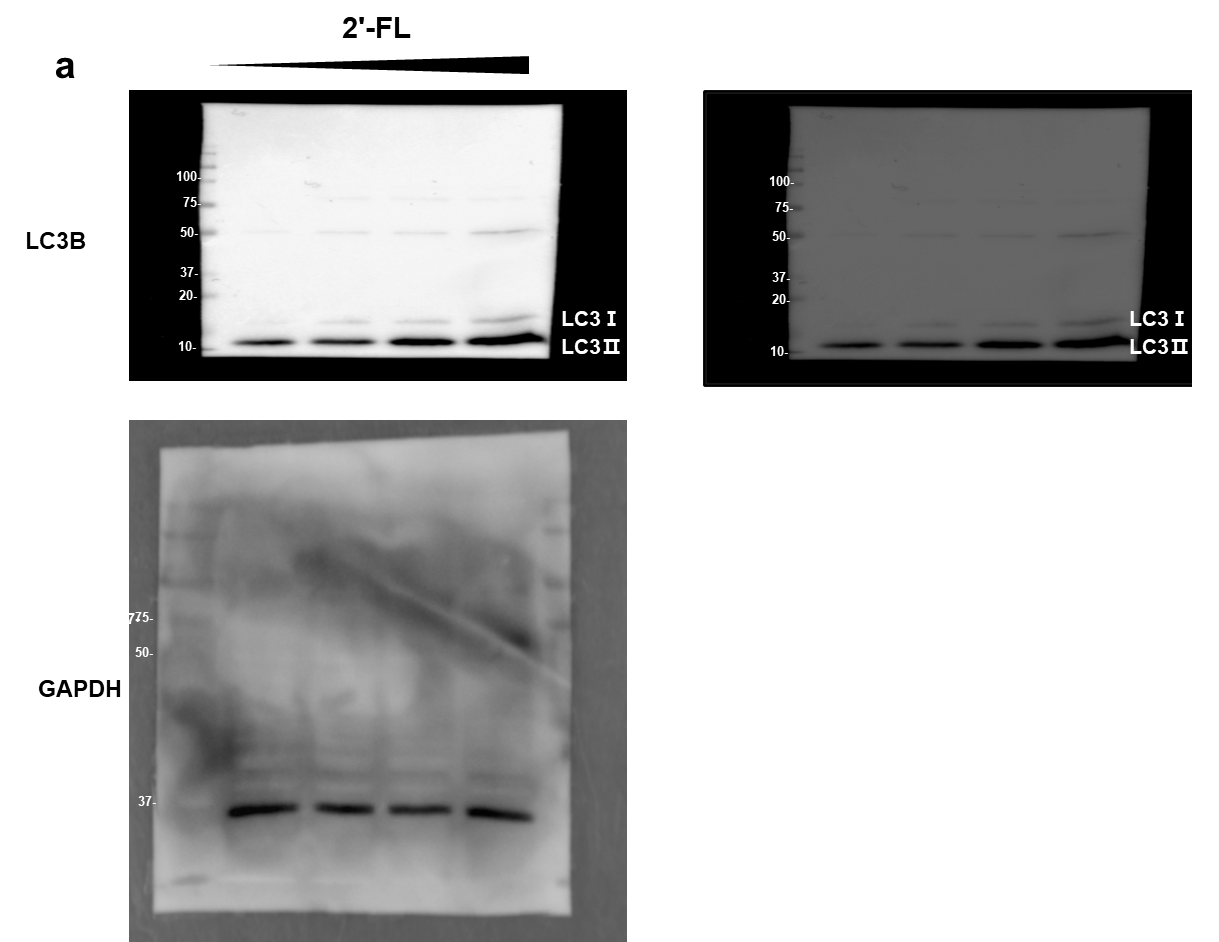
**

**Supplementary Figure S3: 2'-Fucosyllactose induces autophagy via the phosphorylation of AMPK and ULK1.**

**Supplementary Figure S3a.** Full-length images western blot images depicting LC3B (LC3Ⅰ:16kDa, LC3Ⅱ: 14kDa) and GAPDH (37kDa) expression from Figure 3a.

**
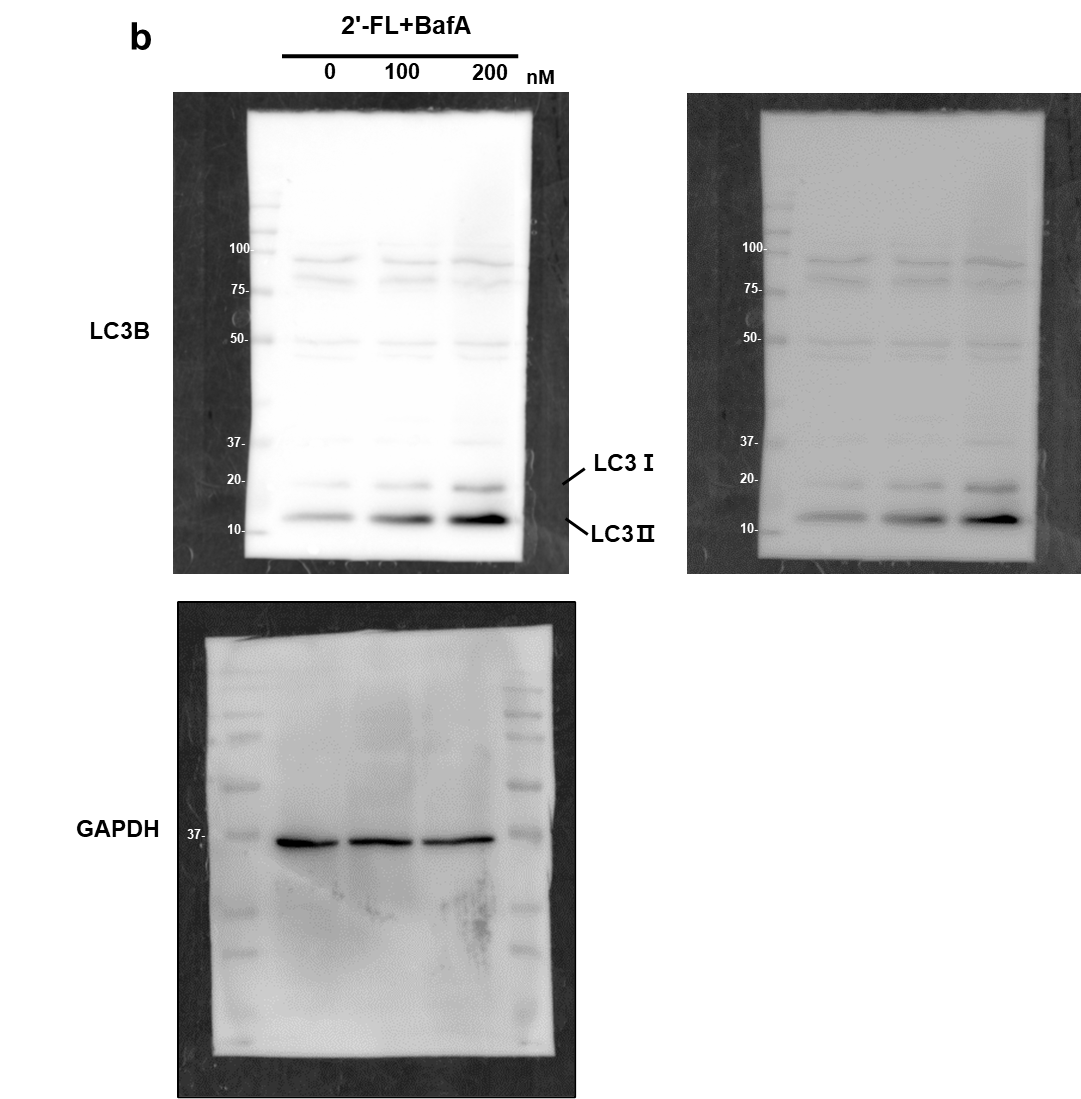
**

**Supplementary Figure S3b.** Full-length images western blot images depicting LC3B (LC3Ⅰ:16kDa, LC3Ⅱ: 14kDa) and GAPDH (37kDa) expression from Figure 3b.

**
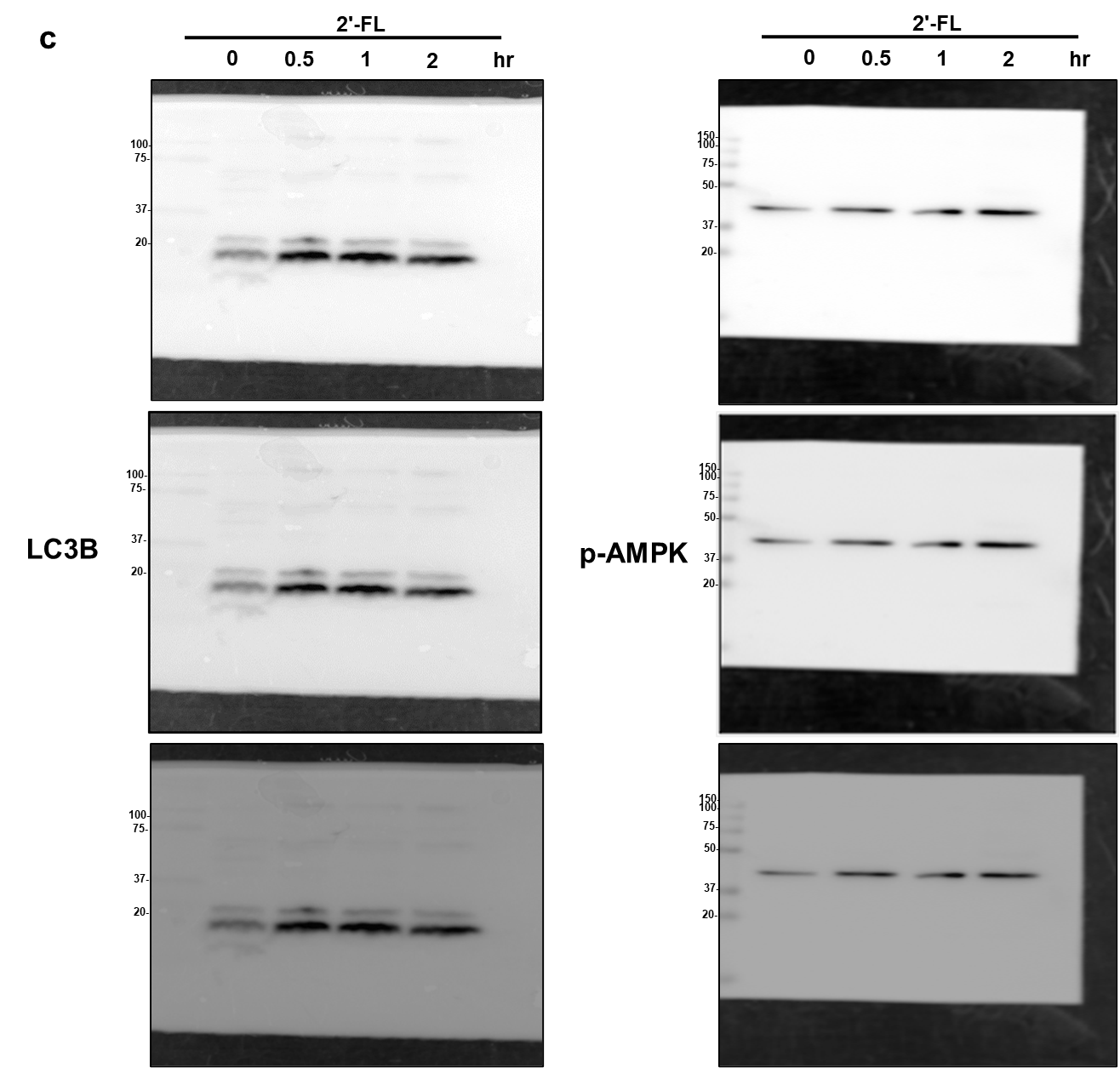
**

**Supplementary Figure S3c.** Full length uncropped WB images depicting LC3B (LC3Ⅰ:16kDa, LC3Ⅱ: 14kDa) and phosphorylated AMPK (62kDa) expression from Figure 3c.

**
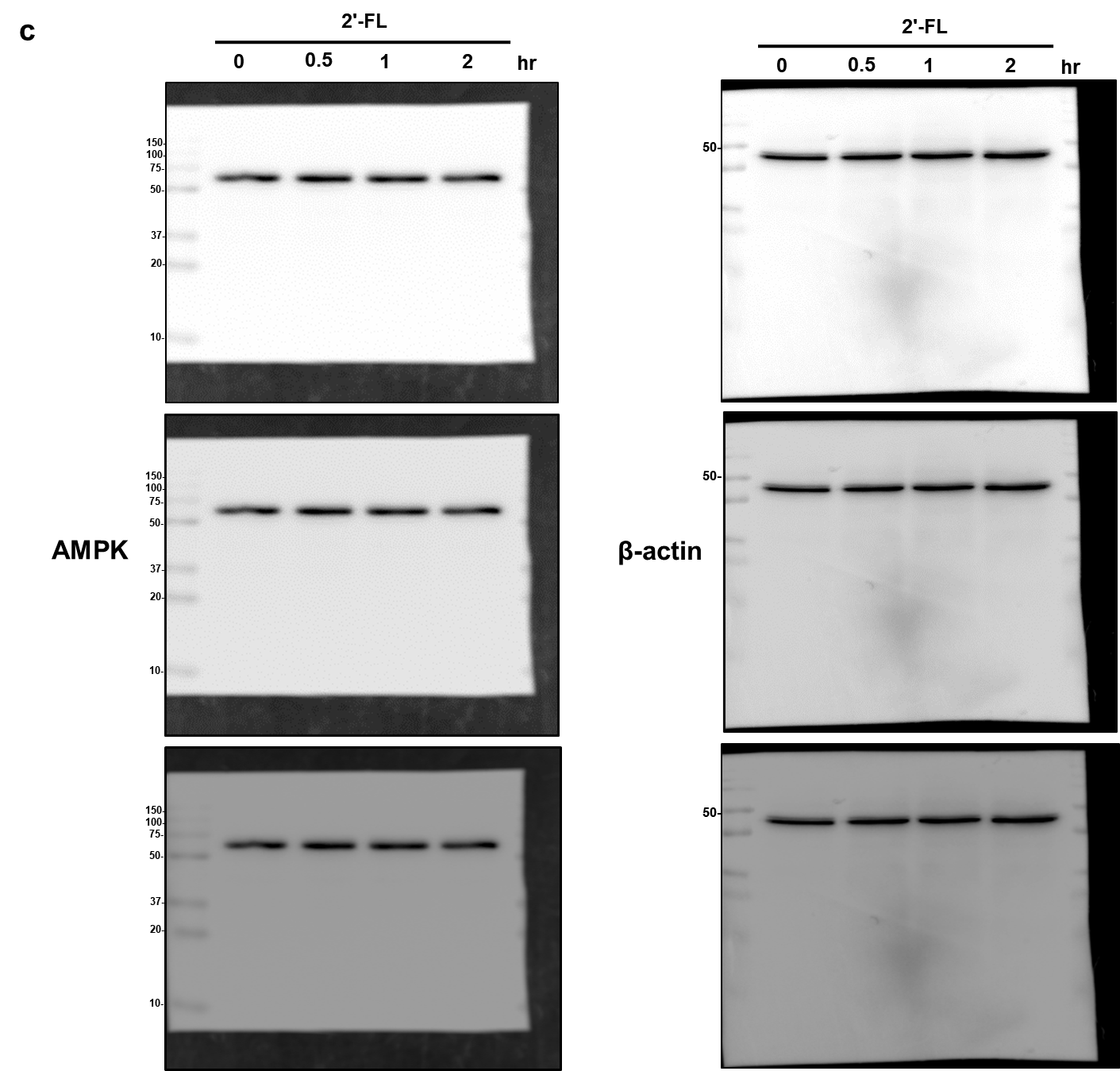
**

**Supplementary Figure S3c.** Full length uncropped WB images depicting total AMPK (62kDa) expression from Figure 3c. The Full-length western blots to accompany β-actin (45kDa) were used as internal controls.

**
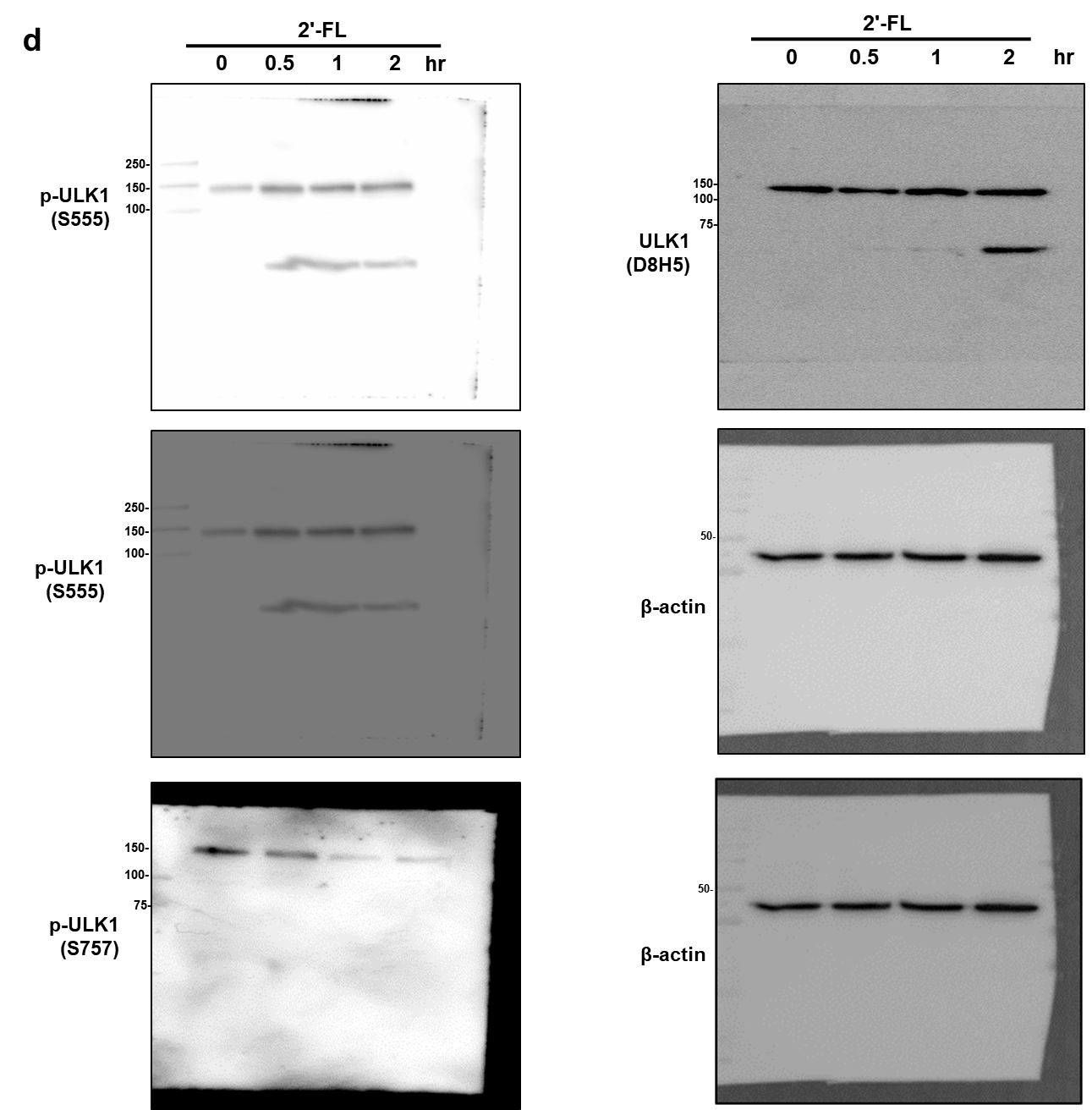
**

**Supplementary Figure S3d.** The same full-length western blot images were used as well as of total and phosphorylated (Ser555 and Ser757:140-150kDa), ULK1 (150kDa) Figure 3d. The Full-length western blots to accompany β-actin (45kDa) were used as internal controls.

**
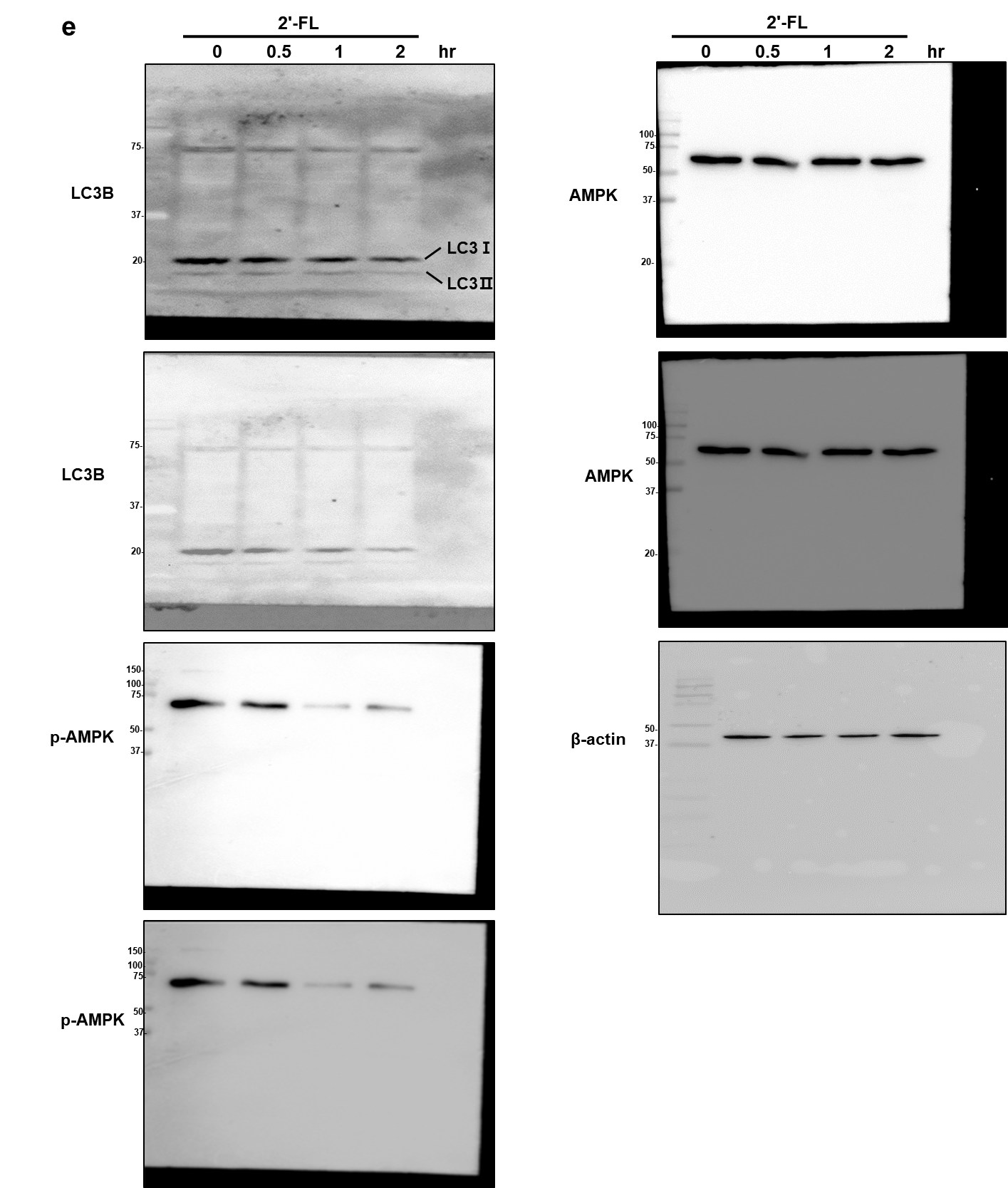
**

**Supplementary Figure S3e.** Full length uncropped WB images depicting LC3B (LC3Ⅰ:16kDa, LC3Ⅱ: 14kDa), total AMPK (62kDa), and phosphorylated AMPK (62kDa) expression from Figure 3e. The Full-length western blots to accompany β-actin(45kDa) were used as internal controls.


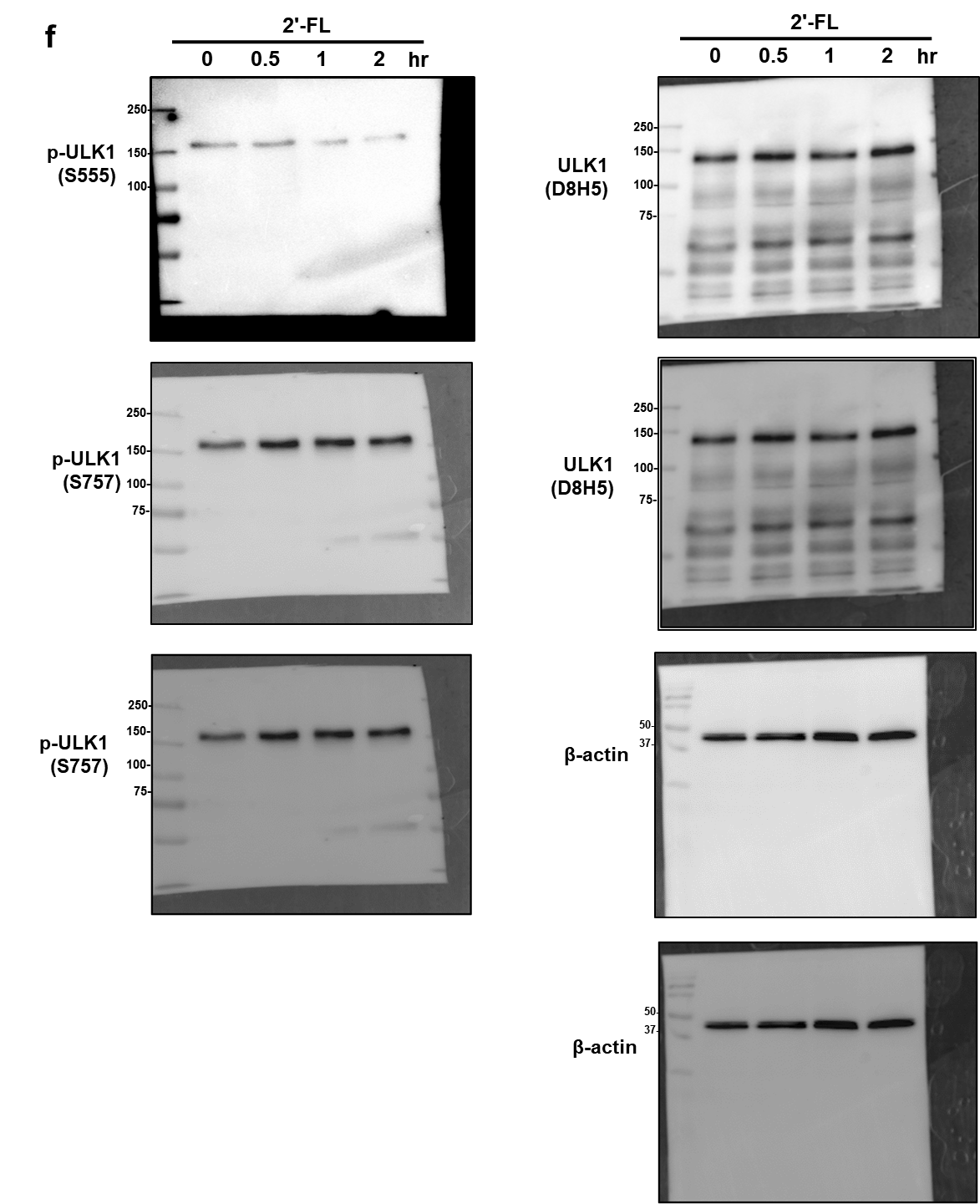


**Supplementary Figure S3f.** The same full-length western blot images were used as well as of total and phosphorylated (Ser555 and Ser757:140-150kDa), ULK1 (150kDa) Figure 3f. Phospho-ULK1 (Ser555) is image marked in red with a molecular weight of 150 kDa was used. The Full-length western blots to accompany β-actin (45kDa) were used as internal controls.

**
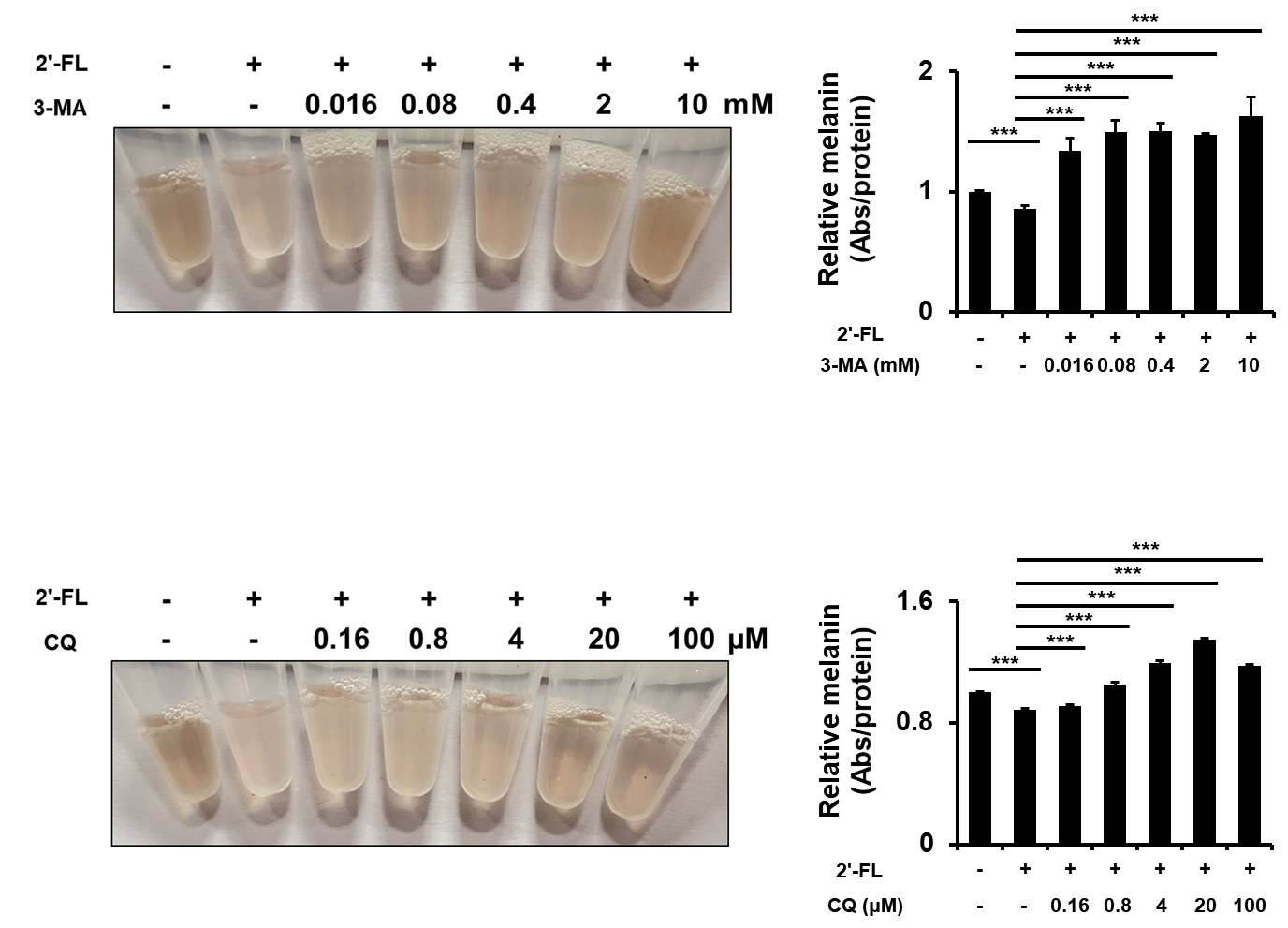
**

**Supplementary Figure S4.** Melanin levels in human MNT-1 cells treated for 5 days with 2'-FL (20 g/L) alone or combined with the autophagy inhibitory drugs at the concentrations stated in the panel.

**Supplementary References**

1 Martin, M. Cutadapt removes adapter sequences from high-throughput sequencing reads. *2011* **17**, 3, doi:10.14806/ej.17.1.200 (2011).

2 Schneider, V. A. *et al.* Evaluation of GRCh38 and de novo haploid genome assemblies demonstrates the enduring quality of the reference assembly. *Genome research* **27**, 849-864, doi:10.1101/gr.213611.116 (2017).

3 Dobin, A. *et al.* STAR: ultrafast universal RNA-seq aligner. *Bioinformatics (Oxford, England)* **29**, 15-21, doi:10.1093/bioinformatics/bts635 (2013).

4 Anders, S., Pyl, P. T. & Huber, W. HTSeq--a Python framework to work with high-throughput sequencing data. *Bioinformatics (Oxford, England)* **31**, 166-169, doi:10.1093/bioinformatics/btu638 (2015).

5 Robinson, M. D., McCarthy, D. J. & Smyth, G. K. edgeR: a Bioconductor package for differential expression analysis of digital gene expression data. *Bioinformatics (Oxford, England)* **26**, 139-140, doi:10.1093/bioinformatics/btp616 (2010).

6 Bolstad, B. M., Irizarry, R. A., Astrand, M. & Speed, T. P. A comparison of normalization methods for high density oligonucleotide array data based on variance and bias. *Bioinformatics (Oxford, England)* **19**, 185-193, doi:10.1093/bioinformatics/19.2.185 (2003).

7 Boo, K. *et al.* Pontin functions as an essential coactivator for Oct4-dependent lincRNA expression in mouse embryonic stem cells. *Nature Communications* **6**, 6810, doi:10.1038/ncomms7810 (2015).

8 Lee, H. J. *et al.* Direct transfer of alpha-synuclein from neuron to astroglia causes inflammatory responses in synucleinopathies. *The Journal of biological chemistry* **285**, 9262-9272, doi:10.1074/jbc.M109.081125 (2010).

9 Hwang, D. *et al.* A data integration methodology for systems biology. *Proceedings of the National Academy of Sciences of the United States of America* **102**, 17296-17301, doi:10.1073/pnas.0508647102 (2005).

10 Ashburner, M. *et al.* Gene ontology: tool for the unification of biology. The Gene Ontology Consortium. *Nature genetics* **25**, 25-29, doi:10.1038/75556 (2000).

11 Kanehisa, M., Goto, S., Sato, Y., Furumichi, M. & Tanabe, M. KEGG for integration and interpretation of large-scale molecular data sets. *Nucleic acids research* **40**, D109-114, doi:10.1093/nar/gkr988 (2012).

12 Huang, D. W., Sherman, B. T. & Lempicki, R. A. Systematic and integrative analysis of large gene lists using DAVID bioinformatics resources. *Nature Protocols* **4**, 44-57, doi:10.1038/nprot.2008.211 (2009).

13 Shannon, P. *et al.* Cytoscape: a software environment for integrated models of biomolecular interaction networks. *Genome research* **13**, 2498-2504, doi:10.1101/gr.1239303 (2003).

14 Stark, C. *et al.* BioGRID: a general repository for interaction datasets. *Nucleic acids research* **34**, D535-539, doi:10.1093/nar/gkj109 (2006).

15 Orchard, S. *et al.* The MIntAct project--IntAct as a common curation platform for 11 molecular interaction databases. *Nucleic acids research* **42**, D358-363, doi:10.1093/nar/gkt1115 (2014).

16 Licata, L. *et al.* MINT, the molecular interaction database: 2012 update. *Nucleic acids research* **40**, D857-861, doi:10.1093/nar/gkr930 (2012).

17 Salwinski, L. *et al.* The Database of Interacting Proteins: 2004 update. *Nucleic acids research* **32**, D449-451, doi:10.1093/nar/gkh086 (2004).

18 Brown, K. R. & Jurisica, I. Unequal evolutionary conservation of human protein interactions in interologous networks. *Genome Biology* **8**, R95, doi:10.1186/gb-2007-8-5-r95 (2007).
